# Habituation and resocialization of a former pet and entertainment chimpanzee: Longitudinal monitoring of physiological and behavioral responses

**DOI:** 10.1007/s10329-026-01240-9

**Published:** 2026-02-02

**Authors:** Paula Serres-Corral, Dietmar Crailsheim, Olga Feliu, Bernat Salelles, Annaïs Carbajal, Manel López-Béjar

**Affiliations:** 1 Department of Animal Health and Anatomy, Universitat Autònoma de Barcelona, 08193 Bellaterra, Spain; 2 Research Department, Fundació Mona, 17457 Girona, Spain

**Keywords:** *Pan troglodytes*, Resocialization, Rescue center, Stress, Cortisol, Social behavior

## Abstract

Environmental changes, such as transfers and social introductions, can be major sources of stress in wild animals. Monitoring the physiological response through glucocorticoids is an effective tool to assess how animals cope with and adjust to these changes. In this study, we followed the habituation and social integration of Suzie, a former pet and entertainment chimpanzee confiscated and transferred to a primate rescue center. Our objectives were to (1) monitor her fecal glucocorticoid metabolite (FGM) levels throughout rehabilitation, and (2) investigate how association-related factors (e.g. number of individuals, ages of association partners) influenced her FGM levels and social behaviors during associations with the resident chimpanzees. A total of 169 fecal samples from Suzie were collected over 10 months and quantified using enzyme immunoassay, along with 38 opportunistic samples from the resident chimpanzees. During association sessions, data on aggressive and affiliative events were recorded. Suzie’s FGM levels ranged from 11.01 to 113.04 ng/g of feces. After a four-month adjustment period (118 days) before returning to basal hormone activity, her FGM concentrations aligned with individual mean levels of the resident chimpanzees (range: 24.19 ± 7.99 to 29.69 ± 8.93 ng/g of feces). The presence of a broker individual during associations impacted the likelihood of affiliative events (*p* < 0.01). Furthermore, early social housing conditions of the resident chimpanzees influenced social dynamics, with affiliative behaviors being more likely to occur while interacting with individuals who were housed in social groups during infancy, and aggressive events being more likely to occur while interacting with those housed alone (*p* < 0.05). Our findings underscore the value of non-invasive hormone monitoring and behavioral assessments to better understand individual experiences.

**Supplementary Information:**

The online version contains supplementary material available at https://doi.org/10.1007/s10329-026-01240-9.

## Introduction

In captivity, animals are exposed to numerous environmental, social, and management factors that may cause stress and negatively affect their health and well-being (Morgan and Tromborg 2007; Carlstead and Shepherdson 2000; Hill and Broom 2009). Additionally, transfers within or between institutions are frequently conducted to preserve genetic diversity or improve living conditions, but the associated transport, introduction to novel environments, and unfamiliar individuals can be significant sources of stress (Powell 2010).

When facing a potential stressful event (physical or psychological), a cascade of behavioral and physiological responses are activated to cope with the stressor (Palme 2019; Palme et al. 2005). The hypothalamic–pituitary–adrenal (HPA) axis is one of the primary stress systems, and involves the release of glucocorticoids (GCs) by the adrenal glands to generate the initial stress response in vertebrates (Reeder and Kramer 2005; Young et al. 2004). As GCs play a central role in the stress response, they can serve as reliable physiological indicators for stress assessments (Palme 2019). Particularly, measuring fecal glucocorticoid metabolites (FGM) has emerged as a valuable non-invasive tool for the assessment of the stress response and overall well-being in wild, free-ranging animals, including nonhuman primates (Novak et al. 2013). In terms of sample type, fecal samples provide a more integrated measure of hormone activity since the last defecation and allow for long-term monitoring without disturbing the animals (Kersey and Dehnhard 2014; Touma and Palme 2005). This methodology is commonly used to evaluate how wild animals in captivity cope with changes in their environment as well as in housing and husbandry conditions, including transfers, relocations, introductions to new social groups or enclosure modifications (Cinque et al. 2017; Serres-Corral et al. 2021; Yamanashi et al. 2016).

Chimpanzees (*Pan troglodytes*) live in multimale-multifemale communities, based on fission–fusion societies, in which members temporarily separate into sub-groups (parties) that split and fuse with other parties depending on ecological and social factors such as food availability (Matsumoto-Oda et al. 1998), predator pressure (Sakura 1994) and/or the presence of receptive females (Anderson et al. 2002; Emery Thompson et al. 2010). Typically, only young sexually mature females leave their natal group in order to emigrate to another neighboring community, while most other inter-community encounters tend to be of aggressive nature and might even lead to fatal outcomes (Kahlenberg et al. 2008; Wilson and Wrangham 2003).

In captivity, natural migration patterns do not occur and group composition as well as alterations to said composition are mostly decided on by the housing organizations and not the chimpanzees themselves, often involving transfers of chimpanzees of different sexes and ages (Crailsheim et al. 2020b). As such, when working on social integration activities such as introducing a new individual into an already established chimpanzee group, it is crucial to consider the risks involved, potential augmentation of stress and the social impact of that new individual on the rest of the social group. Given that considerable stress is often anticipated, ongoing monitoring and adaptive management are essential (Powell 2010). Combining physiological stress indicators with behavioral measures enables a more comprehensive understanding of how animals are coping and adjusting throughout this process.

Previous studies on social integration in captive chimpanzees have shown that both individual and environmental factors influence integration outcomes. Fultz et al. (2022) found that males were more socially interactive and aggressive than females during introductions, that affiliative behaviors were unexpectedly frequent, and that factors such as group size, enclosure type, and age impacted the outcome of an introduction. Similarly, Alford et al. (1995) reported greater aggression during male–male introductions, especially when individuals differed in age or social experience. Furthermore, early life history and social housing conditions shape coping strategies and stress responses during resocialization, with younger and socially enriched individuals showing better adjustment (Reimers et al. 2007; Llorente et al. 2015).

For this study we had the rare opportunity to follow the habituation and social integration of a recently confiscated chimpanzee, Suzie, rescued at the age of 48 years. Suzie had spent her early years as a performer before eventually becoming a pet in a private home, with limited contact with other chimpanzees. Accordingly, our general aim was to evaluate the physiological response of Suzie to her habituation and resocialization at the primate rescue center. Specifically, we aimed to: (1) Longitudinally monitor Suzie’s FGM levels throughout her rehabilitation (i.e., habituation and resocialization); (2) Investigate how various association-related factors (e.g., number of individuals or group type) influenced Suzie’s FGM levels and the occurrence of aggressive or affiliative behaviors during association sessions with resident chimpanzees.

## Materials and methods

### Animals and study site

The present study was conducted between September 2021 and June 2022 at Fundació MONA, a primate rescue and rehabilitation center located in Catalonia, Spain. This center is a member of the European Alliance of Rescue Centers and Sanctuaries (EARS). At the time of this study, two socially stable groups of chimpanzees were housed at MONA (n = 14; Bilinga group: 4 males and 3 females; Mutamba group: 5 males and 2 females) in about 120 m^2^ of indoor and 5640 m^2^ of outdoor enclosure space. From the time of her arrival and throughout her habituation and social rehabilitation, data and fecal samples were collected from Suzie, a 48-year-old chimpanzee rescued after a lifetime in the entertainment industry and as a pet, and opportunistically from her association partners. Suzie had been over-exposed to familiar and un-familiar humans, trained for performances, and although she had been partially living with other chimpanzees, she experienced extensive periods of social isolation, including the decade before her transfer. Upon arrival, she was obese, lacked muscle mass and agility, and had lived in an environment that prevented physical exercise and species-appropriate behaviors.

Following the Center’s procedures, Suzie was first housed in isolation from the other chimpanzees (see Table 1), in a 16 m^2^ indoor and 50 m^2^ outdoor rehabilitation facility, allowing her to hear other chimpanzees, while staying mostly out of visual sight.

**Table 1:** Biographic information of the rescued chimpanzee (Suzie) and the chimpanzees she had contact with during the study

Name	Sex	Year of Birth (Estimated)	Age group	Year of arrival	Predominant social housing condition during infancy	Number of fecal samples
Suzie	Female	1973	Senior	2021	Social	169
Bea	Female	1985	Senior	2012	Social	2
Cheeta	Female	1986	Senior	2015	Single	2
Coco	Female	1994	Adult	2012	Single	7
Waty	Female	1997	Adult	2002	Social	-
Victor	Male	1982	Senior	2006	Single	1
Tom	Male	1985	Senior	2011	Social	23
Tico	Male	1987	Senior	2005	Single	-
Bongo	Male	2000	Adult	2002	Social	-
Nico	Male	2001	Adult	2004	Single	3

### Chronological order of study phases

**Fig. 1:**
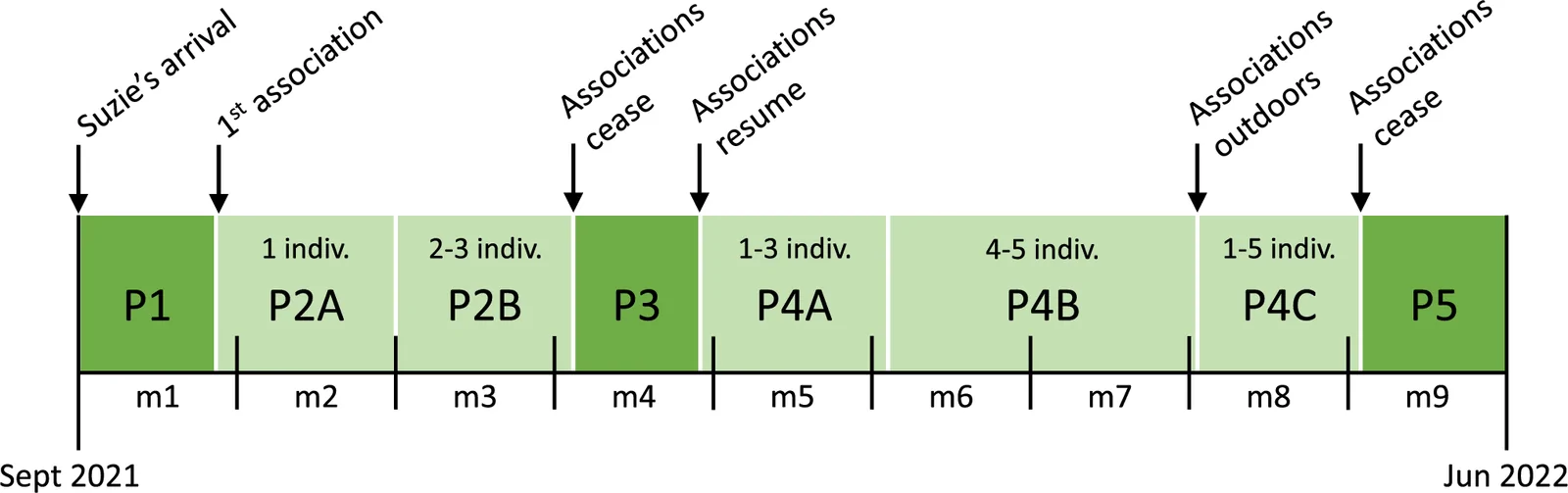
Timeline since the arrival of Suzie to Fundació Mona including: an initial phase in which Suzie was single-housed and had no contact with conspecifics (P1); a phase of one-on-one associations (P2A) followed by associations with two or three individuals (P2B); four weeks in which associations ceased and Suzie remained single-housed (P3); a second phase of associations first with up to three resident chimpanzees (P4A), then increasing the number to four or five individuals at a time (P4B), and finally conducting associations outdoors for the whole day (P4C); and a final phase in which Suzie remained single-housed for four weeks (P5). *m* month; *indiv.* individuals

To evaluate the introduction of Suzie to the novel environment and social group, the study was divided into five phases spanning 39 weeks (Fig. 1):

- **P1:** initial four weeks following her arrival at the center. Suzie was single-housed and had no visual contact with other conspecifics, only indirect auditory contact. Based on the Sanctuary’s rescue and rehabilitation protocols, Suzie was gradually introduced to her new environment, including housing facilities, husbandry team, dietary plan, enrichment program and daily husbandry routines.
- **P2:** first phase of associations with the resident group of chimpanzees (three to seven associations per week). During the first four weeks, sessions consisted of one-on-one encounters (**P2A**). In the following four weeks, individuals were introduced in pairs or groups of three (**P2B**). All sessions occurred in the 50 m^2^ outdoor rehabilitation facility already familiar to Suzie, consisting of two identical adjacent cages (25m^2^ respectively). This outdoor facility is designed to offer varying levels of exposure and safety, with caregivers controlling access between the adjacent cages. Trained staff monitored each session, adjusting exposure, using enrichment to encourage interaction, and stopping sessions as needed.
- **P3**: associations ceased for four weeks. Suzie remained single-housed.
- **P4:** second phase of associations with the resident group (two to five associations per week). During the first month, sessions included one to three chimpanzees of the resident group (**P4A**). Over the following two months, the number of resident chimpanzees was generally increased to four or five individuals (**P4B**). Once Suzie established stronger social bonds, associations with one to five resident chimpanzees were transferred to the naturalistic large outdoor enclosures for the entire day (**P4C**).
- **P5**: final four weeks in which Suzie remained single-housed and had no direct contact with other conspecifics.

The duration of the association sessions varied depending on Suzie’s progression through the integration process: short sessions (< 1 h) were conducted mainly during the initial phases (P2), half-day sessions (1–4 h) occurred throughout most of P2 and P4A, and full-day sessions (> 4 h) took place during P4B and P4C.

### Data collection from association sessions and weather

For each session, we recorded its duration, location, type of contact (direct or indirect) as well as information on cage assignation and chimpanzee combinations. The occurrence of aggressive or affiliative events (see Table S1 for ethogram) between Suzie and the association partners was also recorded, indicating whether each type of social event occurred during the session, without noting frequency or sequence. Additionally, ad libitum notes were taken to document any noteworthy issues and/or advancements in the social integration process. From all this data collected, crucial information was extracted and defined as:

- **Tom presence (Yes/No):** indicates whether Tom participated in the association session. Tom was an adult male who was the first to establish physical affiliative interactions with Suzie and was often used as broker individual, directly or indirectly helping to establish bonds with other chimpanzees.
- **Number individuals associated (1, 2–3, 4–5):** number of resident chimpanzees participating in the association session.
- **Group type (male, female, mixed):** sex composition of the association partners, including all-male, all-female or mixed-sex groups.
- **Age association partners (adult, senior, mixed):** age group of the association partners, including adults, seniors, or a mix of both age groups.
- **Housing conditions infancy (social, solitary, mixed):** indicates whether the association partners were predominantly housed with conspecifics (social) or alone (solitary) during the first five years of their life (Crailsheim et al. 2020b). The mixed group includes resident chimpanzees from both social and solitary housing backgrounds.
- **Aggressive events (No/Yes):** occurrence of one or more aggressive events during the association session.
- **Affiliative events (No/Yes):** occurrence of one or more affiliative events during the association session.

Additionally, daily weather data were obtained from official records. For analysis, days were classified based on mean daily temperature as cold (< 13.2 °C) or warm (> 13.2 °C).

### Fecal sample collection and steroid extraction

Fecal samples from Suzie were collected from her individual indoor enclosure during the daily morning cleaning routines and were immediately frozen at − 20 °C until steroid extraction. Throughout P4, feces were collected generally twice a week, including days with and without associations. Additionally, during the first phase of associations (P2), feces from other chimpanzees were collected opportunistically (see Table 1). These fecal samples were always collected on the day after said individual participated in an association session, thus representing the hormonal activity of the association day. To individualize the fecal samples for the resident group of chimpanzees, non-toxic shredded wax crayons (Giotto be-bè®, Fila Iberia S.L., Spain) were used in small amounts as indigestible markers. Different colors were assigned, and a piece of shredded wax crayon of the corresponding color was mixed in or hidden inside a food item and was given only to the chimpanzees that were taking part in the association with Suzie for that day. The next morning, fecal samples containing wax crayon were collected during the morning cleaning routine of the group enclosures and immediately frozen at − 20 °C.

Fecal samples were dried in an oven at 60 °C for 24 to 48 h, then attached fibrous material (i.e., hay and leaves) was removed and samples were pulverized. Then, 300 mg of powder were extracted with 3 mL 80% methanol. In samples < 300 mg, a proportion of 1 mL 80% methanol for every 100 mg of feces was maintained. The mixture was vortexed for 30 min and then centrifuged at 7.750 × g for 15 min. The supernatant was transferred into 1.5 mL microcentrifuge tubes and frozen at − 20 °C until steroid analysis.

### Steroid analysis

Concentrations of FGM were analyzed with a cortisol enzyme immunoassay (EIA) kit (Neogen® Corporation, Ayr, UK). Cross-reactivity of the Cortisol EIA kit is as follows: prednisolone (47.4%), cortisone (15.7%), 11-deoxycortisol (15.0%), prednisone (7.83%), corticosterone (4.81%), 6ß-hydroxycortisol (1.37%), 17-hydroxyprogesterone (1.36%), deoxycorticosterone (0.94%), progesterone (0.06%), and all other steroids (< 0.06%).

The EIA was biochemically validated by verifying precision, specificity, accuracy and the low limit of quantification (LLOQ) of the assay. Fecal extracts from Suzie were pooled for assay validation. Precision was assessed through intra- and inter-assay coefficients of variation (CV), averaging 8.73% and 19.90%, respectively. Specificity was confirmed through a linearity of dilution test by diluting the sample pool at multiple rations (neat to 1:20) with EIA buffer (Pearson’s correlation test between obtained and expected FGM concentrations: R^2^ = 0.99, *p* < 0.001). Accuracy was evaluated with the spike-and-recovery test by adding known volumes of the sample pool to known volumes of different concentrations of standard cortisol solution (0.4, 1 and 2 ng/mL). The mean recovery percentage (± SD) from the spike-and-recovery test was 112.12 ± 6.10%. The LLOQ of the assay was 0.032 ng FGM/ml of fecal extract, determined by identifying the lowest concentration analyzed with acceptable levels of precision and accuracy.

### Data analysis

Statistical analysis of the data and graphical representation were conducted with R software (R-project, Version 4.3.0, R Development Core Team, University of Auckland, New Zealand) and Graph Pad Software Inc. (GraphPad Prism, version 8.0.2; Graph Pad Software Inc., San Diego, CA, USA). Hormone data were evaluated by visual inspection (i.e., histograms and Q-Q plots) and the Shapiro–Wilk normality test, and then log-transformed to improve distribution. The statistically significant level was settled at a *p* value < 0.05 in all tests performed.

#### Monitoring FGM concentrations in Suzie throughout her rehabilitation

Individual peak and baseline FGM concentrations were determined through an iterative process in which FGM concentrations greater than the mean + 2 standard deviations (SD) were systematically eliminated until no values exceeded that cut-off (mean + 2 SD). All values above the cut-off were then classified as peaks.

A generalized linear model (GLM) with a Gaussian distribution and identity link function was built to evaluate the impact of study phase (i.e., P1 to P5) on overall FGM concentrations. The Walds Chi-Square (χ^2^) test was used to evaluate the marginal significance of the fixed factor (i.e., study phase), and Tukey-adjusted post hoc comparisons were conducted to investigate significant differences between phases. A peak FGM concentration from P3 (60.02 ng/g feces) was excluded from the GLM analysis as it reflected the hormonal activity of the only day in P3 when Suzie was associated with two resident chimpanzees, making it unrepresentative of her single-housed status during P3. However, this concentration was included when calculating the adjustment period for Suzie to return to basal hormonal activity, determined by identifying the first full month with one or less peaks of FGM concentrations (Fazio et al. 2020).

#### FGM concentrations in the resident group of chimpanzees

Descriptive statistics were used to calculate the individual mean (± SD) FGM concentrations for the resident chimpanzees. For Tom, since more fecal samples were regularly collected (see Table 1), the same iterative process previously described for Suzie was applied to determine peak and baseline FGM concentrations. Peak FGM values were excluded from Tom’s individual mean calculation.

#### Influence of association-related factors on Suzie’s FGM levels and social behaviors

Only hormonal and behavioral data from the association phases (P2 and P4) were included in the statistical analysis. Time was included as a random factor in all models, measured in months since Suzie’s arrival. For hormonal data, generalized linear mixed models (GLMMs) with Gaussian distribution and identity link function were employed to evaluate the effects of different predictors on FGM concentrations. Similarly, GLMMs with a binomial distribution and logit link function were used to assess the influence of various predictors on the occurrence of aggressive behavior (0: No/1: Yes) and affiliative behavior (0: No/1: Yes) on association days. For all models, analysis of residuals was conducted through visual inspection of residuals plots and χ^2^ tests were used to evaluate the marginal significance of fixed factors. When statistically significant, Tukey-adjusted post hoc comparisons were conducted to investigate differences between levels of the fixed factors.

Model 1 evaluated the effect of association days (Yes/No) on FGM concentrations. A subset of the database, including only association days, was then used for further analysis. Using this subset, a second set of models was built to assess the effect of Tom’s presence during associations. Specifically, Model 2A assessed the impact of Tom’s presence (Yes/No) on FGM levels, while Models 2B and 2C examined its effect on occurrence of aggressive and affiliative behaviors, respectively. Additionally, Models 3 and 4 explored whether the occurrence of aggressive behavior (Yes/No) and affiliative behavior (Yes/No) on association days influenced Suzie’s FGM concentrations, respectively. All models (Model 1–5) included the interaction between fixed factors and phase (P2, P4) to assess phase-specific responses.

Finally, Models 5, 6 and 7 evaluated the effects of association-related factors on FGM concentrations and on the likelihood of occurrence of aggressive behavior and affiliative behavior, respectively. Fixed factors included the number of individuals associated (1, 2–3, 4–5), group type (male, female, mixed), age of association partners (adult, senior, mixed), housing conditions of association partners during infancy (social, single, mixed) and weather (warm, cold). Multicollinearity between fixed factors was assessed by calculating the variance inflation factor (VIF). For each response variable, model selection was based on Akaike’s Information Criterion corrected for small samples (AICc), comparing 32 candidate models, including the null model (Tables S2-S4).

## Results

### Monitoring FGM concentrations in Suzie throughout her rehabilitation

**Fig. 2:**
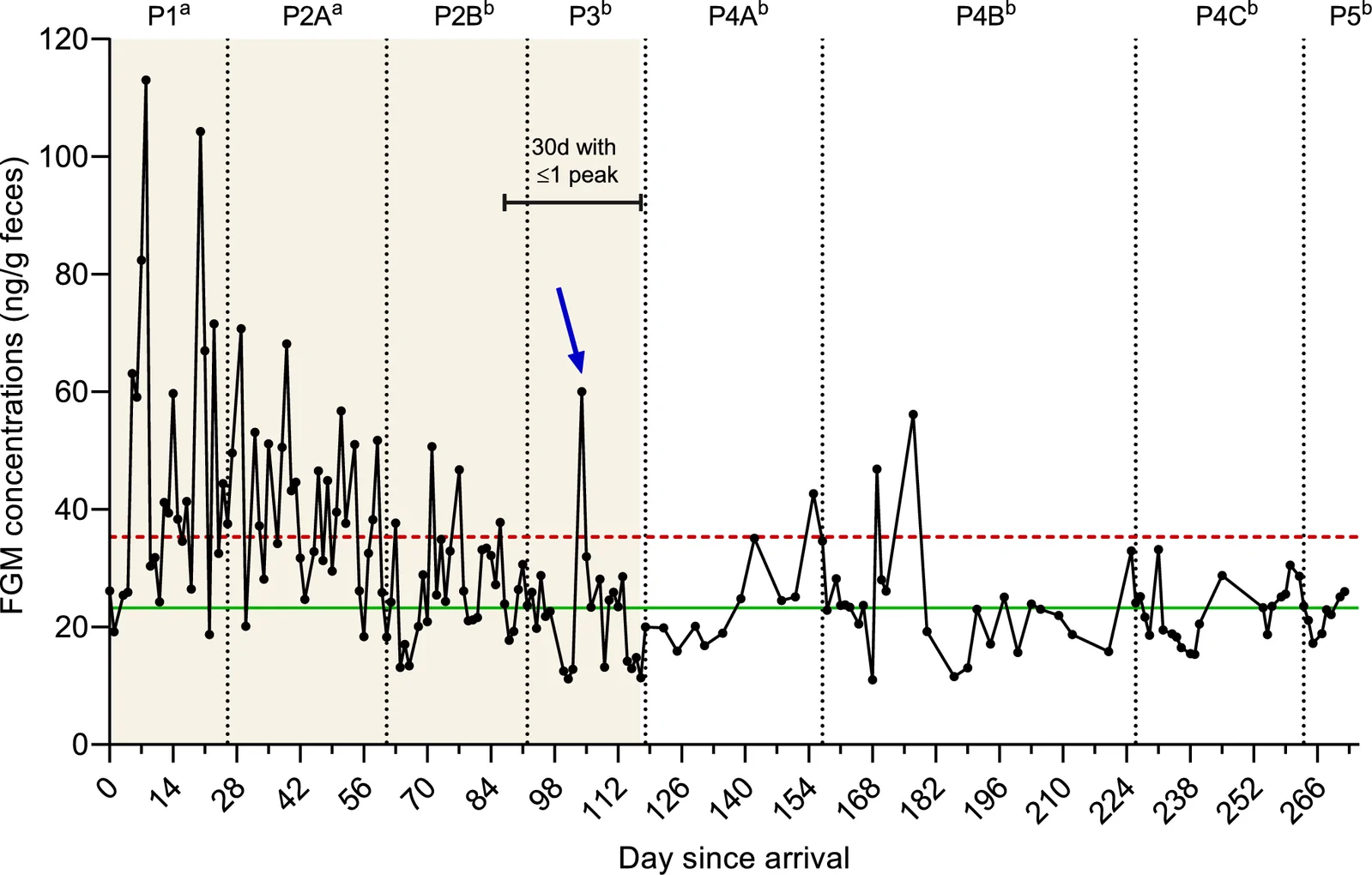
Longitudinal fecal glucocorticoid metabolites (FGM) profile of Suzie following her arrival at the rescue center. The horizontal solid line (green) represents the mean of baseline FGM concentrations. The horizontal dashed line (red) symbolizes the cut-off (mean + 2SD) with all values above the line classified as peaks. The vertical dotted lines (black) show the different study phases (P1 to P5). The shading area shows the adjustment period of Suzie (defined as 30 consecutive days with one or less peaks of FGM). The arrow denotes the only day in P3 when an association took place and was related to a marked peak of FGM. Different lower-case letters (a,b) indicate significant differences (*p* < 0.05) across study phases

Levels of FGM in Suzie ranged from 11.01 to 113.04 ng/g of feces. Study phase had a significant effect on overall FGM concentrations (χ^2^ = 99.05, df = 7, *p* < 0.001). Post hoc pairwise comparisons revealed higher overall FGM levels in P1 and P2A compared to all the other phases (P2B to P5) (*p* < 0.01) (Fig. 2). Based on Suzie’s longitudinal hormone profile, her adjustment period before returning to basal hormonal activity was of approximately four months (118 days).

### FGM concentrations in the resident group of chimpanzees

In the resident group of chimpanzees, FGM concentrations ranged between 12.90 and 101.01 ng/g of feces with an average (± SD) concentration of 33.08 ± 19.50 ng/g of feces. When assessing FGM levels independently for each chimpanzee, individual means (± SD) ranged from 24.19 ± 7.99 to 29.69 ± 8.93 ng/g of feces (Figure S1). In the case of Tom, three peak FGM concentrations were identified (peak mean ± SD = 92.07 ± 7.89 ng/g of feces) and excluded from the calculation of his individual mean.

### Influence of association-related factors on Suzie’s FGM levels and social behaviors

For Models 5 to 7, all VIFs were below 2 (range: 1.02–1.91), indicating that none of the fixed factors were correlated. Results of the Type II Wald χ^2^ tests (Models 1 to 7) are summarized in Table S5. No significant differences were found in Suzie’s FGM concentrations between association and resting days (Model 1; *p* > 0.05). Furthermore, the presence of Tom (Model 2A) and the occurrence of aggressive (Model 3) or affiliative events (Model 4) during association days also did not impact Suzie’s FGM concentrations (*p* > 0.05). As expected, FGM concentrations were significantly higher during P2 compared to P4 across all models (*p* < 0.01).

**Fig. 3:**
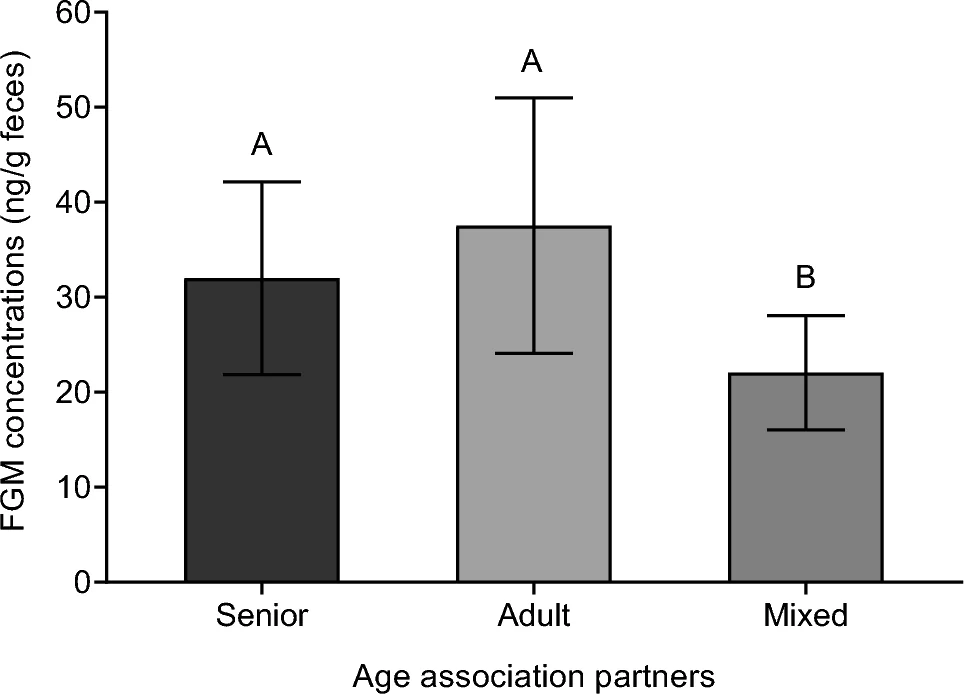
Suzie’s mean (± SD) fecal glucocorticoid metabolites (FGM) concentrations during association days clustered by the age group of the association partners. Different upper-case letters represent significant differences (*p* < 0.01) between groups

Based on AICc model selection, the best candidate model explaining the variability in FGM levels during association days (Model 5) included the number of individuals she associated with and the age group of those chimpanzees. While the number of individuals showed only a trend towards significance (*p* = 0.07), the age group of the chimpanzees significantly influenced Suzie’s FGM levels (*p* < 0.01). Post hoc analysis revealed that Suzie exhibited significantly lower FGM levels on days when she was associated with mixed-age groups compared to days with only adults or seniors (*p* < 0.01) (Fig. 3).

**Fig. 4:**
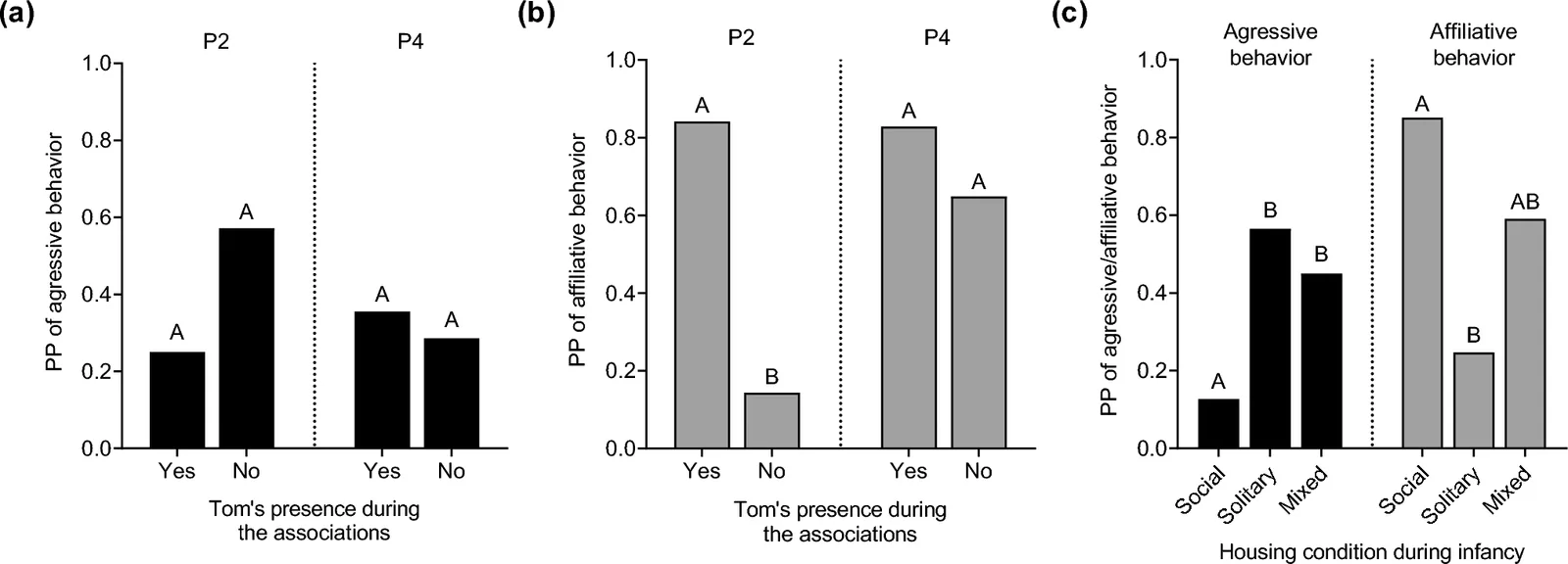
Predicted probabilities (PP) of aggressive and affiliative behaviors occurring on association days as a function of (**a**, **b**) the presence of Tom stratified by phase (P2 and P4), and of (**c**) housing conditions of the association partners during infancy. Different upper-case letters (A, B, AB) denote significant differences (*p* < 0.05) between groups within each phase in graphs (**a**) and (**b**) and within each type of behavior in graph (**c**)

Tom’s presence did not influence the likelihood of aggressive events occurring during associations, regardless of phase (Model 2B; *p* > 0.05) (Fig. 4a). Conversely, it significantly impacted the likelihood of the occurrence of affiliative behaviors, with the effect varying by phase (Model 2C; *p* > 0.01). Post hoc comparisons showed that affiliative behaviors were more likely to occur on days when Tom was present, but only during P2 (*p* < 0.01) (Fig. 4b). Furthermore, AICc model selection identified the best-fit models explaining the likelihood of occurrence of aggressive (Model 6) and affiliative (Model 7) behaviors as those including the housing conditions of the association partners during infancy, with significant effects in both models (*p* < 0.05). Post hoc tests revealed that affiliative behaviors were more likely when association partners had been housed in social groups during infancy compared to solitary housing (*p* < 0.01). In contrast, the likelihood of aggressive events was higher when association partners had been housed alone during infancy compared to social or mixed conditions (*p* < 0.05) (Fig. 4c).

## Discussion

In the present study, we successfully monitored the physiological response of Suzie, a rescued elderly chimpanzee, during her arrival and habituation to the center Fundació MONA. Furthermore, we employed physiological and behavioral metrics to document and evaluate her introduction process into the social group of resident chimpanzees. To this end, we assessed how several association-related factors influenced Suzie’s FGM levels and the occurrence of aggressive and affiliative events during associations with the resident chimpanzees (i.e., her future group members).

It is well established that events such as transfers and introductions to novel environments can be major sources of stress, often requiring weeks or even months to fully acclimate (Dickens et al. 2010; Novak et al. 2013; Powell 2010). Many examples exist in the literature documenting the physiological response to these changes of environment in wildlife species, including non-human primates [e.g., orangutans (Fink et al. 2022; Mendonça et al. 2016); gorillas (Fowler et al. 2022); macaques (Cinque et al. 2017); mandrills (Woodruff et al. 2023)]. In chimpanzees, previous research has shown that relocation and social changes can elicit elevated stress responses that gradually decline with habituation, with individual history and social context strongly influencing recovery patterns (Reimers et al. 2007; Yamanashi et al. 2016). In our study, although pre-transfer samples were not available to assess hormonal changes following transfer, it is likely that hormone levels measured prior to Suzie’s rescue would not have represented a true physiological baseline due to her prolonged social isolation and deprived conditions. Nevertheless, in line with previous studies, Suzie’s longitudinal hormone profile showed a gradual decrease in FGM peaks from her arrival at the rescue center until she had physiologically adjusted to the novel environment. Statistical evaluation of mean FGM levels across study phases further confirmed this, with FGM concentrations in P1 and P2A being significantly higher than in later phases. In addition, from P2B onwards, Suzie’s mean FGM concentrations were within the same range (20–30 ng FGM/g feces) to those found in the other chimpanzees residing in the rescue center, indicating that her adrenocortical activity had stabilized and aligned to levels typical of the long-term residents.

Inter-individual variability in the adrenocortical response to a given stimulus has been widely documented (Cockrem 2013; Mormède et al. 2007). Factors such as the loss of predictability and control associated with being in a novel environment, along with an individual’s prior experience, play crucial roles in the magnitude of the stress response generated and the time required for habituation (Emery Thompson et al. 2010; Sapolsky 2005). In the case of Suzie, it took approximately four months (118 days) for her to adjust before returning to basal hormonal activity, although her FGM levels were already significantly lower after the first two study phases (i.e., around week nine). This timeframe is longer than what has generally been observed in other studies under similar conditions, including a previous study on chimpanzees. For instance, using the same approach, Fazio et al. (2020) reported average adjustment times of 2–3 months before returning to basal adrenocortical activity in male fishing cats following institutional transfers. Similarly, former laboratory chimpanzees presented elevated FGM levels for one to seven weeks after relocation for rehabilitation purposes (Reimers et al. 2007), and mandrills needed about a month to physiologically acclimate to a pre-release enclosure during a translocation, evidenced by FGM levels decreasing over time (Woodruff et al. 2023). Among large mammals, Indian rhinoceroses exhibited stress responses to translocation of variable duration lasting for up to nine weeks (Capiro et al. 2014). In contrast, Asian elephants showed much longer adjustment periods, with FGM concentrations remaining significantly elevated for up to seven months post-relocation (Fanson et al. 2013). These studies highlight that habituation to novel environments is not only species-specific but also highly dependent on individual factors.

Suzie’s prolonged adjustment period may be explained by her previous life experience, including long-term social isolation, which likely compromised her ability to cope appropriately with the stress of relocation to the primate rescue center. Supporting this idea, Reimers et al. (2007) found that former research chimpanzees subjected to earlier maternal separation and longer periods of social isolation exhibited greater and more prolonged stress responses to relocation than their younger conspecifics. Furthermore, in addition to the relocation itself, which can be seen as a fixed unique event, after the first habituation phase, Suzie was participating in social association sessions with varying combinations of social partners on almost a daily basis, which could also likely lead to a prolonged adjustment time.

Behavioral interactions with conspecifics can be substantial sources of stress (Creel 2001), especially in relation to social dominance and hierarchy, as widely studied in primates (Muller and Wrangham 2004; Sapolsky 2005). In the same line, social interactions between unfamiliar or socially inexperienced individuals may be highly stressful (Feliu et al. 2022; Wittig et al. 2016). In our study, during the first phase in which social introductions were ceased and Suzie remained single-housed (P3), a notable peak in FGM levels was observed on the single day Suzie was associated with two resident chimpanzees (see Fig. 2). This peak further highlights the validity of our FGM measurements, demonstrating that they reliably reflect the physiological response to social stressors. Nevertheless, except for that peak, no significant differences were found in Suzie’s FGM levels between association and resting days during the two phases of socialization with the resident group of chimpanzees (P2 and P4). Furthermore, Suzie’s FGM concentrations did not differ based on the occurrence of aggressive or affiliative events on association days.

Typically, aggressive or agonistic interactions have been linked to increases in GCs secretion (Creel 2001; Muller and Wrangham 2004; Yamanashi et al. 2016), whereas positive affiliative interactions have been related with reducing GCs levels and mitigating stress (Jacobs et al. 2014; Wittig et al. 2008; Wooddell et al. 2017). The lack of a clear relationship between the occurrence of these behaviors during associations and Suzie’s FGM levels suggests that such events may not have been sufficient to elicit a consistent physiological response detectable through feces. Since behavioral data were recorded as presence/absence of affiliative or aggressive events per session, it is possible that a more detailed measure considering the frequency or intensity of social interactions might have revealed subtler relationships between behavior and FGMs. Additionally, other daily external factors not accounted for may have also influenced Suzie’s FGM concentrations and potentially masked any effects of punctual aggressive or affiliative events during the associations.

The presence of Tom (an adult male chimpanzee who managed to establish affiliative contact with Suzie early on and, as a result, was spending the most time together with Suzie) during the social introductions with the resident chimpanzees proved essential for her integration into the social group. This observation was made by experienced professionals involved in her rehabilitation and was further supported by our findings. While aggressive events happened independently of Tom’s presence during associations, affiliative behaviors were significantly more likely to occur when Tom was present, particularly during the first phase of social introductions (P2). However, these behavioral trends did not translate into physiological differences, as no differences were found on Suzie’s FGM levels between association days with or without Tom. Numerous studies have emphasized the importance of social support in primate species and how close social bonds can help ameliorate stress (Novak et al. 2013; Sapolsky 2005). The presence or interaction with bond partners during stressful events can have a buffering effect, resulting in lower GCs levels or facilitating a faster return to basal hormone activity after such events, compared to situations where social support is lacking (Engh et al. 2006; Wittig et al. 2016, 2008; Young et al. 2014). In Suzie’s case, we expect Tom’s presence to have likely played a similar role, enabling her to have a smoother integration into the social group, although this effect was not reflected in her adrenocortical activity.

Several association-related factors were explored with regards to Suzie’s variability in HPA axis activity and behavior during association days. While we found a significant effect of the age group of the resident chimpanzees on Suzie’s FGM levels, this was restricted to lower FGM levels on days with groups of mixed ages. However, Suzie’s FGM levels were comparable on days when she was associated with only senior or only adult chimpanzees, suggesting that the differences found may lack biological significance. More importantly, we found that the likelihood of aggressive and affiliative events occurring on association days were best explained by the housing conditions of the resident chimpanzees during infancy. Specifically, affiliative behaviors were more prevalent on association days with resident chimpanzees that had been housed with conspecifics during their early years, whereas days of association with chimpanzees housed alone during their development were more likely to involve aggressive events. This is consistent with findings suggesting that early life conditions, such as predominant social housing during infancy, i.e., the first 5 years of their lives, can have a significant and lasting impact on the chimpanzees’ social capacities and preferences (Crailsheim et al. 2020b). Previous studies on former pet and entertainment chimpanzees suggest that limited time with conspecifics restricts opportunities to observe and practice both affiliative and aggressive behaviors (Crailsheim et al. 2020a; Kalcher et al. 2008; McEwen 2003). Based on these findings, we would expect resident chimpanzees who were housed with conspecifics during infancy to engage more readily in affiliative interactions, creating a social environment that promotes Suzie’s integration and reduces the likelihood of aggressive events. On the contrary, resident chimpanzees that who were not housed with conspecifics during infancy, might be worse equipped to handle tense and potentially stressful or even dangerous situations such as being confronted with an unfamiliar or little familiar chimpanzee, here Suzie.

## Conclusions

In the present study, we monitored the physiological response of Suzie, a former pet and entertainment chimpanzee, during her habituation and socialization after her arrival at Fundació MONA, a primate rescue and rehabilitation center. Our findings indicate that Suzie required a longer time to physiologically adjust to the new environment than what has typically been reported on chimpanzees and other mammals. This longer adjustment period may be explained by her previous life experiences, including prolonged social isolation. Furthermore, we found that the presence of a broker individual functioning as initial bonding partner and social bridge to other future group members during the association sessions, may facilitate the integration into the social group. Finally, we demonstrated the impact that early social housing conditions may have on the social dynamics during said associations.

While this work provides valuable insights on the habituation and resocialization of rescued chimpanzees, its focus on a single subject warrants caution when generalizing the results, as it may not fully capture the inter-individual variability in stress responses. Nevertheless, this study highlights the value of monitoring the physiological response to relocations and emphasizes the importance of including behavioral assessments for a more comprehensive understanding of each individual’s experience. When evaluating the stress response and overall welfare in similar situations, the unique circumstances and individuality of each case should be accounted for to implement effective rehabilitation and resocialization strategies.

## Supplementary Information

Below is the link to the electronic supplementary material.

Supplementary file1 (DOCX 79 KB)

## Data Availability

The data that support the findings of this study are available from the corresponding author upon reasonable request.
