# Supplementary material for: Habituation and resocialization of a former pet and entertainment chimpanzee: Longitudinal monitoring of physiological and behavioral responses
Source: Primates. 2026 Feb 2;67(2):187–97. doi: 10.1007/s10329-026-01240-9 (PMC12957016; doi:10.1007/s10329-026-01240-9)
Supplement: Supplementary file 1 — Supplementary file1 (DOCX 79 KB) [file 10329_2026_1240_MOESM1_ESM.docx]

**Supplementary Information:**

**Habituation and resocialization of a former pet and entertainment chimpanzee: Longitudinal monitoring of physiological and behavioral responses**

Paula Serres-Corral^1^, Dietmar Crailsheim^2^, Olga Feliu^2^, Bernat Salelles^1^, Annaïs Carbajal^1,*^, Manel López-Béjar^1,*^

^1^ Department of Animal Health and Anatomy, Universitat Autònoma de Barcelona, 08193 Bellaterra, Spain

^2^ Research Department, Fundació Mona, 17457 Girona, Spain

* Authors have contributed equally to the direction of this study

Corresponding author: Paula Serres-Corral (paula.serres@uab.cat)

**Table S1.** Ethogram used by the head caregivers during the integration process to score the presence or absence of affiliative and agonistic events involving Suzie and the other chimpanzees.

| **Affiliative events** | This includes all socio-positive behaviors occurring between one or several individuals present in the same enclosure. | | |
| --- | --- | --- | --- |
|  | **Social Play** |  | Ludicrous behavior between two or more individuals punctually accompanied by indicators of play (e.g., play face, laughter, gallop, cartwheels and pirouettes). This activity may include a game of tag, catch and grab limbs or objects, tickling, muzzling, rough-and-tumble play. These interactions do not require permanent physical contact. |
|  | **Grooming** |  | Body-cleansing behavior from one individual to another (includes mutual grooming), performed with the upper extremities and/or with the mouth. |
|  | **Embrace** |  | An individual hugs or attempts to hug another individual with one or both arms in the absence of an agonistic event or agonistic indicators such as aggressive or submissive mimics. This behavior can occur while stationary or while in movement. |
| **Agonistic events** | This includes all agonistic interactions directed to one or several individuals present in the same enclosure, that s is being answered by either an agonistic or submissive response. | | |
|  | **Aggression** |  | Behaviors relating to the agonistic/display threat, direct aggression, wounding, chasing, displacement and/or resource appropriation (including object and social resources). It can be accompanied by vocalizations. The performing individual is typically exhibiting more dominant and impressive body postures to appear bigger and more threatening. Movements are broad and often contain drumming, swaggering, stomping etc. |
|  | Explanation of **Submissive action/reaction** |  | Behaviors indicating fear or subordinate intentions toward one or several other chimpanzees. The chimpanzee shows signs of fear directed at another individual in an agonistic context (often accompanied by a fear grin). It may include approaching and presenting vulnerable body parts to appease or attempt to avoid or flee from an individual displaying aggressive behavior. It can include actions such as genital presenting, extending limbs, hand to mouth, finger to mouth, running away, avoiding proximity, and is typically accompanied by a mimic indicating fear and vocalizations such as yelling, screaming, crying, alarm calling or pant-grunting. |


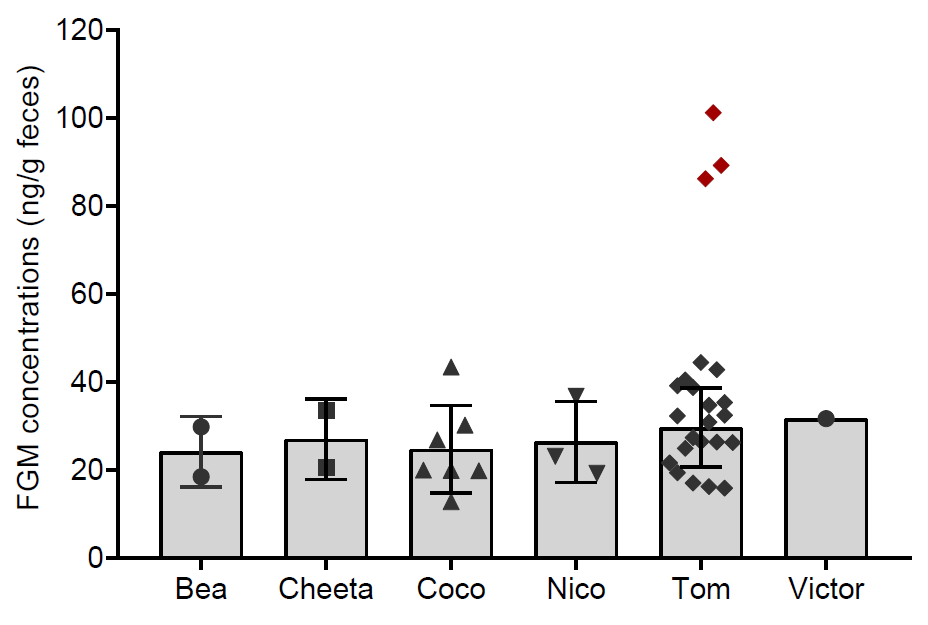


**Figure S1.** Individual mean (±SD) fecal glucocorticoid metabolites (FGM) concentrations for the chimpanzees of the resident group. For Tom, three peak FGM concentrations were identified (shown in red) and excluded from the calculation of his individual mean (±SD). In the case of Victor, only one fecal sample could be collected, thus the value of this sample is shown instead of an individual mean.

**Table S2.** Ranking of candidate models based on Akaike’s Information Criterion corrected for small samples (AICc) to evaluate the effects of association-related factors on fecal glucocorticoid metabolite (FGM) concentrations. Fixed factors included the number of individuals associated (N Ind ass), group type (Grp typ), age of association partners (Age ass ind), housing conditions of association partners during infancy (inf) and weather (Wth). Time (measured in months) was included as a random factor in all candidate models. Only models accounting for up to 0.95 of the cumulative weight are shown, along with the null (0) and full (1) models for reference.

| **Model** | **Age ass ind** | **Grp typ** | **Hsn inf** | **N Ind ass** | **Wth** | **df** | **AICc** | **delta** | **weight** |
| --- | --- | --- | --- | --- | --- | --- | --- | --- | --- |
| **10** | **+** |  |  | **+** |  | **7** | **-99.40** | **0.00** | **0.28** |
| 6 | + |  |  |  |  | 5 | -98.90 | 0.45 | 0.22 |
| 17 | + |  |  |  | + | 6 | -97.30 | 2.09 | 0.10 |
| 23 | + |  |  | + | + | 8 | -97.20 | 2.17 | 0.09 |
| 13 | + | + |  |  |  | 7 | -96.60 | 2.70 | 0.07 |
| 15 | + | + |  |  |  | 7 | -95.20 | 4.12 | 0.04 |
| 0 |  |  |  |  |  | 3 | -95.00 | 4.38 | 0.03 |
| 26 | + | + |  |  | + | 8 | -94.90 | 4.48 | 0.03 |
| 21 | + | + |  | + |  | 9 | -94.60 | 4.71 | 0.03 |
| 19 | + | + |  | + |  | 9 | -94.60 | 4.79 | 0.03 |
| 27 | + | + |  |  | + | 8 | -93.40 | 6.00 | 0.01 |
| 7 |  |  |  |  | + | 4 | -93.30 | 6.07 | 0.01 |
| 3 |  |  |  | + |  | 5 | -92.40 | 6.99 | 0.01 |
| … |  |  |  |  |  |  |  |  |  |
| 1 | + | + | + | + | + | 12 | -87.30 | 12.02 | 0.00 |

**Table S3.** Ranking of candidate models based on Akaike’s Information Criterion corrected for small samples (AICc) to evaluate the effects of association-related factors on the likelihood of occurrence of aggressive behaviors. Fixed factors included the number of individuals associated (N Ind ass), group type (Grp typ), age of association partners (Age ass ind), housing conditions of association partners during infancy (inf) and weather (Wth). Time (measured in months) was included as a random factor in all candidate models. Only models accounting for up to 0.95 of the cumulative weight are shown, along with the null (0) and full (1) models for reference.

| **Model** | **Age ass ind** | **Grp typ** | **Hsn inf** | **N Ind ass** | **Wth** | **df** | **AICc** | **delta** | **weight** |
| --- | --- | --- | --- | --- | --- | --- | --- | --- | --- |
| **4** |  |  | **+** |  |  | **4** | **101.20** | **0.00** | **0.24** |
| 8 |  |  | + |  | + | 6 | 101.40 | 0.24 | 0.21 |
| 12 | + |  | + |  |  | 6 | 102.80 | 1.66 | 0.10 |
| 14 |  |  | + |  | + | 5 | 103.20 | 1.98 | 0.09 |
| 20 |  |  | + |  | + | 7 | 103.30 | 2.15 | 0.08 |
| 25 | + |  | + |  | + | 7 | 104.70 | 3.54 | 0.04 |
| 3 |  |  |  |  | + | 4 | 105.00 | 3.80 | 0.04 |
| 18 | + |  | + |  | + | 8 | 105.00 | 3.84 | 0.03 |
| 13 | + |  |  |  | + | 6 | 105.30 | 4.14 | 0.03 |
| 19 | + |  | + |  | + | 8 | 105.40 | 4.25 | 0.03 |
| 24 | + | + | + |  |  | 8 | 107.10 | 5.87 | 0.01 |
| 29 | + |  | + |  | + | 9 | 107.10 | 5.93 | 0.01 |
| 11 |  |  | + |  | + | 5 | 107.30 | 6.07 | 0.01 |
| 30 | + |  | + |  | + | 9 | 107.40 | 6.24 | 0.01 |
| 26 | + |  | + |  | + | 7 | 107.40 | 6.25 | 0.01 |
| 10 | + |  | + |  |  | 6 | 107.60 | 6.39 | 0.01 |
| … |  |  |  |  |  |  |  |  |  |
| 0 |  |  |  |  |  | 2 | 107.60 | 6.40 | 0.01 |
| … |  |  |  |  |  |  |  |  |  |
| 1 | + | + | + | + | + | 11 | 111.60 | 10.44 | 0.00 |

**Table S4.** Ranking of candidate models based on Akaike’s Information Criterion corrected for small samples (AICc) to evaluate the effects of association-related factors on the likelihood of occurrence of affiliative behaviors. Fixed factors included the number of individuals associated (N Ind ass), group type (Grp typ), age of association partners (Age ass ind), housing conditions of association partners during infancy (inf) and weather (Wth). Time (measured in months) was included as a random factor in all candidate models. Only models accounting for up to 0.95 of the cumulative weight are shown, along with the null (0) and full (1) models for reference.

| **Model** | **Age ass ind** | **Grp typ** | **Hsn inf** | **N Ind ass** | **Wth** | **df** | **AICc** | **delta** | **weight** |
| --- | --- | --- | --- | --- | --- | --- | --- | --- | --- |
| **4** |  |  | **+** |  |  | **4** | **97.50** | **0.00** | **0.33** |
| 13 | + |  | + |  |  | 6 | 98.10 | 0.66 | 0.23 |
| 14 |  |  | + |  | + | 5 | 99.50 | 2.00 | 0.12 |
| 26 | + |  | + |  | + | 7 | 100.20 | 2.78 | 0.08 |
| 12 |  | + | + |  |  | 6 | 101.50 | 4.01 | 0.04 |
| 8 |  |  | + | + |  | 6 | 101.90 | 4.46 | 0.04 |
| 19 | + |  | + | + |  | 8 | 102.10 | 4.66 | 0.03 |
| 24 | + | + | + |  |  | 8 | 102.70 | 5.28 | 0.02 |
| 25 |  | + | + |  | + | 7 | 103.70 | 6.22 | 0.02 |
| 20 |  |  | + | + | + | 7 | 104.10 | 6.63 | 0.01 |
| 30 | + |  | + | + | + | 9 | 104.50 | 7.01 | 0.01 |
| 6 | + |  |  |  |  | 4 | 104.80 | 7.36 | 0.01 |
| 0 |  |  |  |  |  | 2 | 104.80 | 7.39 | 0.01 |
| … |  |  |  |  |  |  |  |  |  |
| 1 | + | + | + | + | + | 11 | 109.40 | 11.93 | 0.00 |

**Table S5.** Summary of type II Wald Chi-Square (χ²) tests for the generalized linear mixed models (GLMMs) analyzing Suzie’s fecal glucocorticoid metabolites (FGM) levels and the likelihood of occurrence of aggressive and affiliative behaviors during associations (response variables) in relation to several fixed factors. Month was included as random factor in all models to account for the effect of time.

| **Response variable** | **Model** | **Effect** | **χ²** | **df** | ***p* value** |
| --- | --- | --- | --- | --- | --- |
| FGM | Model 1 | Association day | 0.93 | 1 | 0.335 |
|  |  | Phase | 7.62 | 1 | **0.006** |
|  |  | Association day*Phase | 2.82 | 1 | *0.093 .* |
|  | Model 2A | Tom presence | 0.16 | 1 | 0.690 |
|  |  | Phase | 8.43 | 1 | **0.004** |
|  |  | Tom presence*Phase | 0.08 | 1 | 0.774 |
|  | Model 3 | Aggressive behavior | 0.16 | 1 | 0.685 |
|  |  | Phase | 10.16 | 1 | **0.001** |
|  |  | Aggressive behavior*Phase | 1.24 | 1 | 0.265 |
|  | Model 4 | Affiliative behavior | 1.18 | 1 | 0.278 |
|  |  | Phase | 10.08 | 1 | **0.002** |
|  |  | Affiliative behavior*Phase | 3.19 | 1 | *0.074* *.* |
|  | Model 5 | Number individuals associated | 5.36 | 2 | *0.069* *.* |
|  |  | Age association partners | 12.54 | 2 | **0.002** |
| Aggressive behavior | Model 2B | Tom presence | 1.91 | 1 | 0.167 |
|  |  | Phase | 0.02 | 1 | 0.882 |
|  |  | Tom presence*Phase | 2.20 | 1 | 0.138 |
|  | Model 6 | Housing conditions infancy | 8.52 | 2 | **0.014** |
| Affiliative behavior | Model 2C | Tom presence | 4.87 | 1 | **0.027** |
|  |  | Phase | 0.05 | 1 | 0.824 |
|  |  | Tom presence*Phase | 7.84 | 1 | **0.005** |
|  | Model 7 | Housing conditions infancy | 9.24 | 2 | **0.010** |

Significant differences (*p* < 0.05) are denoted in bold and tendencies (*p* < 0.1) in italic.
